# Synaptic input and temperature influence sensory coding in a mechanoreceptor

**DOI:** 10.3389/fncel.2023.1233730

**Published:** 2023-09-12

**Authors:** Jens-Steffen Scherer, Kevin Sandbote, Bjarne L. Schultze, Jutta Kretzberg

**Affiliations:** ^1^Department of Neuroscience, Computational Neuroscience, Faculty VI, University of Oldenburg, Oldenburg, Germany; ^2^Department of Neuroscience, Cluster of Excellence Hearing4all, Faculty VI, University of Oldenburg, Oldenburg, Germany; ^3^Research Center Neurosensory Science, University of Oldenburg, Oldenburg, Germany

**Keywords:** leech, touch cell, action potential, spike initiation zone, dendritic integration, spike latency, neuronal compartmentalization, temperature

## Abstract

Many neurons possess more than one spike initiation zone (SIZ), which adds to their computational power and functional flexibility. Integrating inputs from different origins is especially relevant for sensory neurons that rely on relative spike timing for encoding sensory information. Yet, it is poorly understood if and how the propagation of spikes generated at one SIZ in response to sensory stimulation is affected by synaptic inputs triggering activity of other SIZ, and by environmental factors like temperature. The mechanosensory Touch (T) cell in the medicinal leech is an ideal model system to study these potential interactions because it allows intracellular recording and stimulation of its soma while simultaneously touching the skin in a body-wall preparation. The T cell reliably elicits spikes in response to somatic depolarization, as well as to tactile skin stimulation. Latencies of spikes elicited in the skin vary across cells, depending on the touch location relative to the cell’s receptive field. However, repetitive stimulation reveals that tactilely elicited spikes are more precisely timed than spikes triggered by somatic current injection. When the soma is hyperpolarized to mimic inhibitory synaptic input, first spike latencies of tactilely induced spikes increase. If spikes from both SIZ follow shortly after each other, the arrival time of the second spike at the soma can be delayed. Although the latency of spikes increases by the same factor when the temperature decreases, the effect is considerably stronger for the longer absolute latencies of spikes propagating from the skin to the soma. We therefore conclude that the propagation time of spikes from the skin is modulated by internal factors like synaptic inputs, and by external factors like temperature. Moreover, fewer spikes are detected when spikes from both origins are expected to arrive at the soma in temporal proximity. Hence, the leech T cell might be a key for understanding how the interaction of multiple SIZ impacts temporal and rate coding of sensory information, and how cold-blooded animals can produce adequate behavioral responses to sensory stimuli based on temperature-dependent relative spike timing.

## 1. Introduction

Understanding neuronal computation with diverse inputs remains a fundamental challenge in neuroscientific research. In both vertebrates and invertebrates there are many neurons that have more than one site where action potentials can be elicited. Examples include human dorsal root ganglia neurons (Nascimento et al., 2022), pyramidal cells in rats and mice (Golding and Spruston, 1998; Larkum et al., 2001; Shu et al., 2007; Goaillard et al., 2020), crustacean stomatogastric ganglion neurons (Vedel and Moulins, 1977; Moulins et al., 1979; Meyrand et al., 1992; Städele and Stein, 2016; Städele et al., 2018; Daur et al., 2019), neurons in *Lymnaea stagnalis* (Haydon and Winlow, 1982), guinea pigs (Wong et al., 1979; Wood and Mayer, 1979), alligator Purkinje cells (Llinas and Nicholson, 1971), as well as neurons in locust (O’Shea, 1975; Heitler and Goodman, 1978), *Aplysia* (Tauc and Hughes, 1963; Evans et al., 2003), and the leech (Calabrese, 1980; Melinek and Muller, 1996; Baccus et al., 2001; Crisp, 2009). The ubiquity of neurons with multiple spike initiation zones (SIZ) across species suggests that their functional benefits outweigh the metabolic costs of higher channel densities (Kole et al., 2008; Lorincz and Nusser, 2008; Schmidt-Hieber et al., 2008; Günay et al., 2015; Nascimento et al., 2022). The arguably biggest advantage of having multiple SIZ is that the functional compartmentalization adds to a neuron’s computational power and flexibility (Clarac and Cattaert, 1999; Larkum et al., 2022). The impact of dendritic spikes on dendritic integration and compartmentalization was studied for decades (Larkum et al., 2001; Stuart and Spruston, 2015; Francioni and Harnett, 2022). For example, dendritic spikes in L5 pyramidal neurons can interact with spikes generated in the axon to produce various spiking patterns (Larkum et al., 2001; Stuart and Spruston, 2015; Francioni and Harnett, 2022). In *Lymnaea stagnalis*, the three axonal SIZ of pleural ganglion neurons can act independently of each other or in concert, depending on the synaptic input to the soma (Haydon and Winlow, 1982).

**Figure 1:**
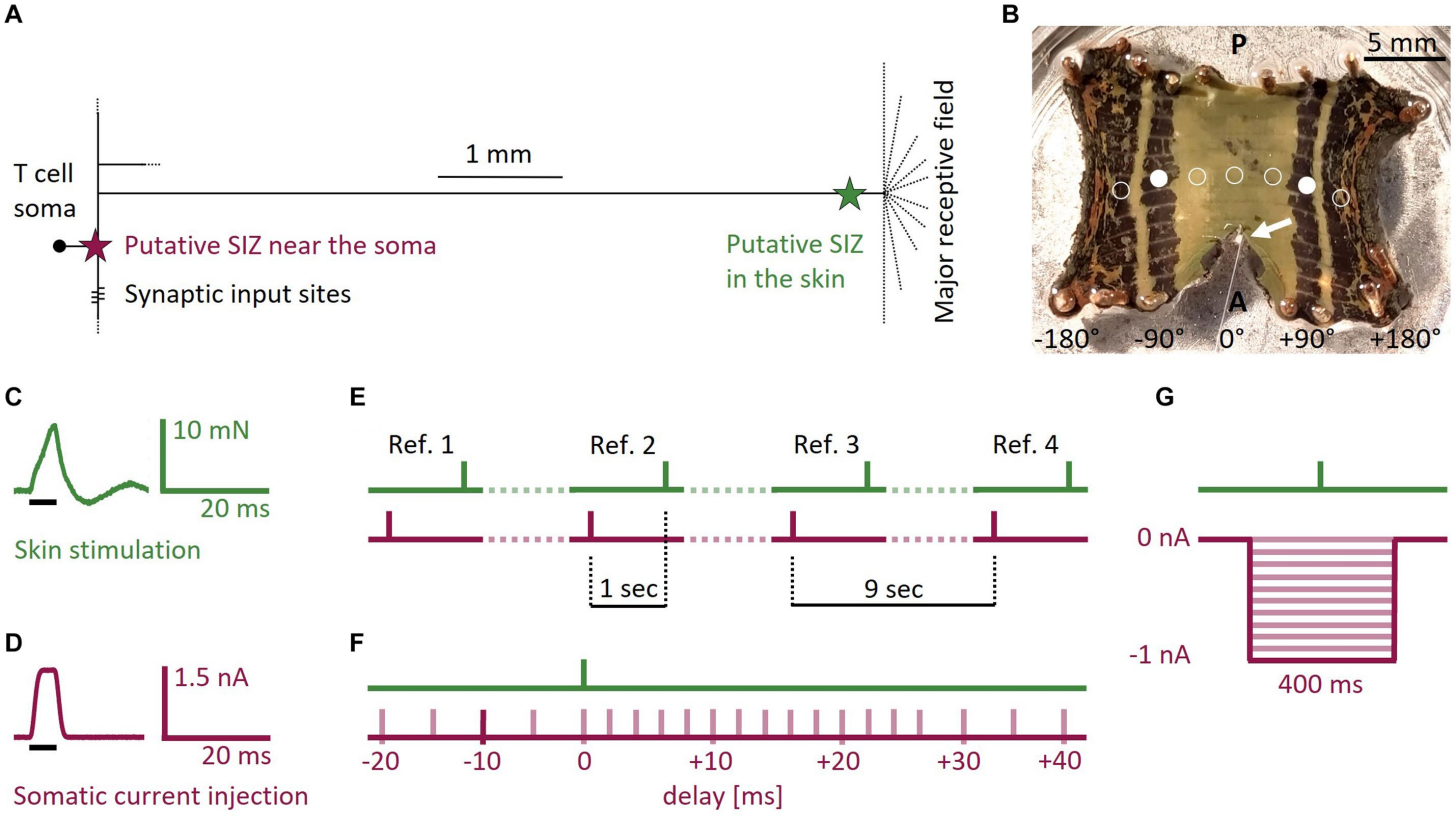
T cell anatomy, body-wall preparation, and stimulus protocols. **(A)** Sketch of the T cell anatomy with putative locations of two SIZ, one near the soma and one in the skin. **(B)** Photograph of a body-wall preparation. Stretching the connective in anterior direction made the ganglion (white arrow) accessible for intracellular recordings while stimulating the skin tactilely. Most T cells spiked reliably when stimulated at +90° or −90° (filled white circles, *n* = 29 preparations). If the *standard tactile pulse* did not trigger at least one spike at this location, we changed the position of the stimulation to ±45° (*n* = 17), ±135° (*n* = 10), or 0° (*n* = 2), shown with empty circles. A, Anterior; P, Posterior. **(C)** *Standard tactile pulse stimulus*. The green line shows the applied force recorded via the stimulation device. The black bar indicates the timing of the 5 ms long command input. The feedback control of the stimulation device caused the actual touch to over- and undershoot the intended rectangular stimulus force, consistently leading to a peak at 8 mN and an oscillation that subsided after 20 ms. **(D)** *Standard electrical pulse stimulus*. The red line shows the injected current, recorded by the amplifier. The black bar indicates the timing of the 5 ms long command input. **(E)** Four *reference* stimuli throughout the *SIZ combination* protocol served as baseline for the expected spike counts and latencies. In the *references* the responses to the *standard electrical pulse* and the *standard tactile pulse* were one second apart and therefore did not interact. Each of the three periods between the *references* (dotted lines) contained seven of the pairs of one *tactile* and one *electrical pulse* from the *SIZ combination* protocol. The *tactile pulses* were separated by one second, yielding a separation time of 9 s between *references*. **(F)** Schematic overview of the *SIZ combination* protocol. We stimulated the skin with the *standard tactile pulse* **(C)** in combination with depolarizing the soma with the *standard electrical pulse* **(D)**. We systematically varied the relative timing between both pulses from −20 to +40 ms delay. In the example shown by the dark red bar, the *electrical pulse* was injected 10 ms before the skin was touched. The whole stimulation protocol consisted of 21 pairs of a *tactile* and an *electrical pulse* that was applied between 20 ms before (negative numbers) and 40 ms after (positive numbers) the *tactile pulse*. These 21 pairs of pulses (timing shown in by light red bars) were interleaved with four *references* as shown in **(E)**. **(G)** Schematic overview of the *hyperpolarization* protocol. We stimulated the skin with a single *tactile pulse* while hyperpolarizing the soma with current steps of 400 ms duration and amplitudes ranging 0 to −1 nA in steps of −0.1 nA (red traces).

The interaction of multiple SIZ could be particularly relevant for mechanosensory neurons that integrate both sensory and synaptic inputs, like the mechanosensory Touch (T) cell in the leech. This cell encodes tactile stimulation of its receptive field in the skin (Yau, 1976; Blackshaw, 1981; Pinato and Torre, 2000; Titlow et al., 2013). Spike count and first spike latency of tactilely induced T cell spikes depend on the location and intensity of the touch stimulus (Pirschel and Kretzberg, 2016). Additionally, spikes from the skin are susceptible to neuromodulation (Gascoigne and McVean, 1991) and conduction block (Nicholls and Baylor, 1968; Van Essen, 1973; Yau, 1976; Mar and Drapeau, 1996; Cataldo et al., 2005). The high temporal precision of T cell responses indicates temporal encoding of stimulus location (Pirschel and Kretzberg, 2016) and suggests a role of T cells in the local bend reflex, with which leeches bend away locally when being touched (Kristan, 1982). Additionally, T cells receive excitatory and inhibitory synaptic inputs, which both can trigger spikes in isolated ganglia without attached skin (Scherer et al., 2022). This is consistent with the conclusion from prior experimental and modeling studies that a T cell has one SIZ close to the soma that primarily responds to synaptic inputs, in addition to another SIZ close to the skin responding to tactile stimulation (Burgin and Szczupak, 2003; Kretzberg et al., 2007), summarized in Figure 1A. The exact location of these SIZ is unknown. It is also unclear, if tactilely induced spikes interact with excitatory and inhibitory synaptic inputs in the ganglion, or if both compartments work independently of each other.

Besides internal factors like neuronal compartmentalization and the resulting integration of sensory and synaptic inputs, global factors like temperature are known to influence the number, shape and timing of spikes in nervous systems (Hodgkin and Katz, 1949; Robertson and Money, 2012; Eberhard et al., 2015; Marder and Rue, 2021). In the leech, temperature was shown to affect the activation rate and amplitude of I_h_ currents in HN cells (Angstadt and Calabrese, 1989) and to inhibit the Na^+^/K^+^ pump in T cells (Catarsi and Brunelli, 1991). As in other poikilothermic organisms, these temperature changes also influence the behavior in leeches (Elliott and Tullett, 1986; Hitchcock et al., 2017).

In the present study, we intracellularly recorded the membrane potential of the leech T cell while mechanically stimulating the skin. We found that tactilely induced spikes are elicited reliably and – despite their longer propagation time – temporally more precise than spikes evoked by current injection into the soma. The first spike latency of tactilely induced spikes measured in the soma increased systematically during somatic hyperpolarization of the membrane potential. By systematically varying the relative timing of spikes elicited at the two SIZ, we observed that fewer spikes reached the soma when their expected arrival times were close to each other. Additionally, we found that lower temperatures increased the propagation times of spikes elicited in the skin by tactile stimulation, and of spikes triggered by somatic current injection at the central SIZ.

## 2. Materials and methods

### 2.1. Animals and preparation

The datasets of this study originate from in total 26 adult hermaphrodite medicinal leeches (*Hirudo verbana*; 1-3 g; *Biebertaler Leech Breeding Farm*, 35444 Biebertal, Germany), which we kept at room temperature (17-25°C) in 24 L tanks filled with artificial pond water (ocean sea salt diluted with purified water, 1:1000) and under natural daylight cycles. Before and during dissection, we anesthetized the leeches in ice-cold leech saline (115 mM NaCl, 4 mM KCl, 1.8 mM CaCl_2_, 10 mM Glucose, 4.6 mM Tris–maleate, 5.4 mM Tris base, pH 7.4, Muller and Scott, 1981). Body-wall preparations were performed by cutting the skin longitudinally along the dorsal midline, leaving the rest of the segment intact. The body-wall was cut anteriorly and posteriorly of the segment of interest. The resulting rectangular preparation (Figure 1B) was flattened and pinned ventral side up in a Petri dish with silicone elastomer *Sylgard* (Dow Corning Corporation, Midland, MI, United States). Once the skin was pinned and stretched out, the sensilla became visible and were used to identify the five annuli belonging to the midbody segment of interest (Blackshaw et al., 1982). We pulled the connective in anterior direction until the ganglion became accessible for intracellular recordings.

### 2.2. Electrophysiology

We performed intracellular single recordings on the mechanosensory T cells using sharp electrodes filled with 4 M potassium acetate (pH adjusted to 7.4). Electrode resistances ranged from 12 to 35 MΩ (mean = 24.26 MΩ, std = 5.06 MΩ). Electrodes were pulled from borosilicate thin-wall capillaries (TW100F-4, World Precision Instruments Inc., Sarasota, FL, United States) with a P97 Flaming Brown micropipette puller (Sutter Instruments Company, Novato, CA, United States). We performed the recordings using a mechanical micromanipulator type MX-1 (TR 1, Narishige, Tokyo, Japan) and a BA-1 s amplifier (NPI Electronic, Tamm, Germany). We identified the T cells according to their soma location and response patterns (Nicholls and Baylor, 1968). Current was injected into the soma while recording the membrane potential (sample rate 10 or 100 kHz; custom MATLAB software).

### 2.3. Tactile stimulation

The tactile skin stimulation was performed with a Dual-Mode Lever System (300C-l, Aurora Scientific, Canada) with a tip size of 0.79 mm^2^. We stimulated the skin in the third annulus of the segment of interest (Figure 1B). Within this third annulus, the ventral midline was defined as 0° and the lateral edges as −180° and +180°, respectively (Pirschel and Kretzberg, 2016). The default starting position was ±90° (filled white circles), on the ipsilateral side of the recorded cell. Even though predominantly the lateral T cell has its receptive field at this position, usually more than one T cell at the ipsilateral side of the ganglion reliably responded to touch with spikes that could be measured with the recording electrode at the soma (see Supplementary Figure S1 for an example of all three ipsilateral T cells responding to touch at the same position). If no spikes were elicited by the *standard tactile stimulus*, we changed the stimulus location along the third annulus to either ±135°, ±45°, or 0° (empty circles in Figure 1B). Hence, we did not aim for one of the three ipsilateral T cells specifically, even though their anatomy and spiking behavior were shown to differ in isolated ganglia (Meiser et al., 2023).

### 2.4. Temperature control

Temperature manipulations were performed by adding ice to a larger support Petri dish surrounding the smaller experimental Petri dish with the preparation. We either added the ice at the beginning of an experiment and thereby decreased the temperature. Or we used a pre-cooled support Petri dish and let the temperature in the experimental Petri dish increase to room temperature again. Via this approach, the lowest temperature in all experiments was 7.9°C and the highest 23.9°C. We tracked the temperature in the experimental Petri dish using a digital multimeter (PeakTech 2025, Ahrensburg, Germany). The experiments for the main results presented in sections 3.1–3.3 were recorded without temperature manipulation at room temperature between 17°C and 25°C, depending on the season they were recorded in.

### 2.5. Stimulus protocols

We performed experiments with the following protocols that were repeated for at least ten trials, with a 1 s pause between trials. All pulses were identical between preparations and throughout the entire recording time of all experiments.

- ***Standard tactile pulse***: for eliciting single spikes in the skin, we used a standard square command pulse with a duration of 5 ms and an amplitude that was calibrated to reach a peak of 8 mN. Due to the feedback loop of the lever system, the resulting output force applied to the skin deviated from the intended square pulse, as shown in Figure 1C. It oscillated for 20 ms before subsiding. This pulse elicited in different preparations between 1 and 4 spikes.
- ***Standard electrical pulse***: we used a standard square current command pulse with a duration of 5 ms and an amplitude of 1.5 nA (Figure 1D) to elicit a single T cell spike in the SIZ close to the soma (Figure 1A).
- ***References***: we used the *standard tactile* (Figure 1C) and *electrical* (Figure 1D) *stimulation pulses* to elicit spikes in both SIZ. These individual pulse stimuli are referred to as *references* when they were applied with an inter-stimulus-interval of 1 s, because the time between them was long enough to exclude potential mutual effects on spike timing. The responses to these references served as our baseline for the expected spike counts and latencies in response to both types of stimulation. Four *references* (Figure 1E) were integrated into the *SIZ combination* protocol explained below (Figure 1F).
- ***SIZ combination***: this protocol was designed to investigate suprathreshold interactions of the two spike initiation zones (Figure 1F) and consisted of pairs of one *standard electrical pulse* and one *standard tactile pulse* as described before (see *references*). We systematically varied the relative timing between the two stimulation types from −20 ms (somatic current injection before touching the skin) to +40 ms (touching the skin before somatic current injection) to investigate the interaction of spikes from both origins. The inter-stimulus-interval between the pair of pulses was 1 s. In total, we applied 21 pairs of pulses with different time delays, which were interleaved with four *references* (Figure 1E).
- ***Hyperpolarization***: this protocol was designed to study the effects of somatic hyperpolarization on the propagation of spikes from the skin (Figure 1G). We previously showed that T cells receive spontaneous, mostly inhibitory synaptic inputs from the network (Scherer et al., 2022). We mimicked these effects by hyperpolarizing the soma while applying a single *standard tactile pulse* (Figure 1C). The *standard tactile pulse* was applied 200 ms after the onset of a step current with a duration of 400 ms and amplitudes ranging from −0.1 to −1 nA in steps of 0.1 nA (Figure 1G) to investigate a systematic relationship between the hyperpolarization amplitude and first spike latencies of tactilely induced spikes. The inter-stimulus-interval was 2 s.
- ***Temperature***: This protocol was designed to investigate the effects of temperature on spike propagation. For this purpose, we used a modified version of the protocol *SIZ combination* and additionally varied the temperature over trials. Instead of 1.5 nA, the current pulse amplitude was either 1 nA (*n* = 9) or 2 nA (*n* = 5), and the relative timing of tactile and electrical stimulation covered a broader range of time delays than the *SIZ combination* protocol, from −40 to +50 ms. The *standard tactile pulse* was the same as in the *SIZ combination* protocol.

### 2.6. Data analysis

In total, we recorded from *N* = 68 T cells. Recorded data was saved into MATLAB files. Each recording was converted into a HDF5 file format and loaded into Python, using the *SciPy* package. The analysis scripts were written in Python 3.8.15. In a first data preprocessing step we excluded all recordings from the analysis that showed resting membrane potentials more depolarized than −30 mV (*n* = 2), or no reliable spiking in the first of the four *references* of the first trial (*n* = 8). Eventually, we analyzed *n* = 58 recordings (*hyperpolarization*: *n* = 20, *SIZ combination*: *n* = 24, *temperature*: *n* = 14). Spikes were detected using the *findpeaks* function of the *SciPy* package with a minimum peak prominence of 6 mV and a minimum peak distance of 2 ms.

Since the measured input resistances varied considerably between cells and over trials in our dataset, we restricted our analysis of the *hyperpolarization* protocol to the first trial and cells within the inter-quartile range of input resistances measured in the first trial based on a − 0.1 nA stimulation, ranging 9-38 MΩ. This very conservative approach was chosen to prevent that electrodes with broken or clogged tips could bias the recorded membrane potential hyperpolarization in response to negative current injection.

Analysis of repeated *references* revealed that first spike latencies of spikes elicited by tactile skin stimulation, as well as by current injection into the soma increased over the course of the four *references* (Supplementary Figure S2; statistical comparison between responses to first and fourth *reference*: electrical: *z* = 4.88, *p* < 0.01, tactile: *z* = 3.94, *p* < 0.01). The biggest latency change can be observed between the responses to the first and second *reference*, the later responses differ only marginally. To account for this systematic bias, we excluded the responses to the first *reference* from all analyses and matched *references* 2-4 pairwise to their preceding pair of a *tactile* and an *electrical standard pulse* in *SIZ combination*. All analyses concerning the responses to *reference* stimuli were performed based on the responses to the fourth *reference* (sections 3.1 and 3.4).

**Figure 2:**
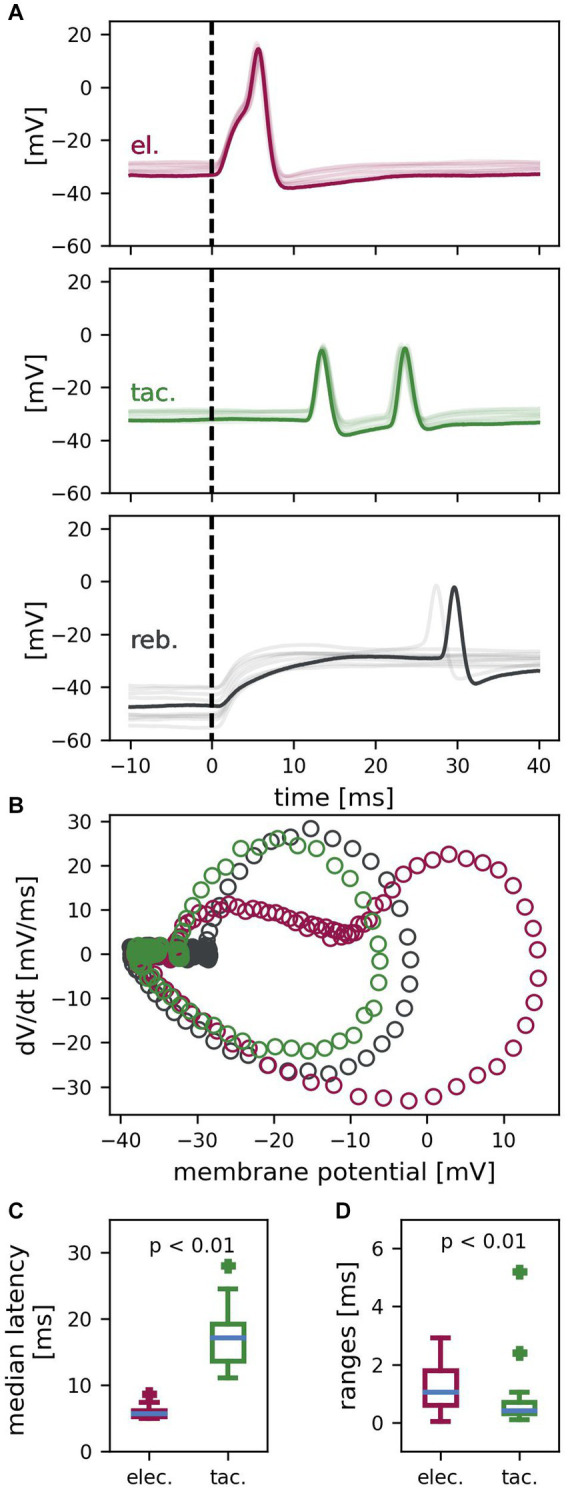
T cell spike responses to different types of stimulation. **(A)** Example recording of one T cell with ten trials superimposed (one example trial in dark color, the other 9 trials in lighter color). Electrically (el., red) elicited spikes, tactilely (tac., green) induced spikes and rebound spikes (reb., gray) differ in latency, reliability, and temporal precision. Dashed vertical lines indicate the start of stimulation with the *standard electrical* or *tactile pulse* or the release of the hyperpolarization, respectively. **(B)** Phase plane trajectories of the spikes of the example trial shown in **(A)**. Trajectories resemble each other, besides the passive response to electrical stimulation (red) shifting the starting points of the steep phase of the change in membrane potential to a more depolarized membrane potential. **(C)** Median latencies of electrically and tactilely induced spikes (*n* = 24 cells, *reference stimulus* #4 in 10 trials per cell). Latencies of tactilely induced spikes are significantly longer than latencies of electrically elicited spikes (*z* = 4.29, *p* < 0.01) and are more variable between cells. **(D)** Latency ranges of repeated responses to electrical vs. tactile stimulation of the same cell (*n* = 24 cells, *reference stimulus* #4 in 10 trials per cell). First spike latencies have a significantly smaller range (and are therefore more precise) for tactilely than for electrically elicited spikes (*z* = 2.8, *p* < 0.01).

The following features were assessed as dependent variables for the statistical analysis:

- **First spike latency** (ms) was defined as the time difference between the first spike peak and stimulus onset of the *standard tactile or electrical pulse*, respectively. For rebound spikes, the latency was defined as time difference between the first spike peak and the offset of the hyperpolarization of −1 nA.
- **Precision** (ms) was defined as the range of first spike latencies in response to repetitive *reference* stimulus presentations. The smaller the range, the higher the precision of the cell.
- **Reliability** (%) was defined as the probability of the occurrence of at least one spike in response to repetitive *reference* stimulus presentations. The higher the probability, the higher the reliability of the cell.
- **Expected spike time difference** (ms) was defined as the expected time difference between spikes elicited via the pair of a *standard tactile* and *electrical pulse*, given the time difference between these pulses (see *SIZ combination*), as well as the different propagation times to reach the recording electrode in the soma for spikes from both SIZ (Figure 1A). For both types of stimulation, we calculated the first spike latency based on the responses to the *references*. E.g., a first spike latency of 6 ms for a spike elicited via current injection and 13 ms for a tactilely induced spike would result in an expected spike time difference of −7 ms if both types of stimulation pulses were applied synchronously. If the current was injected 10 ms before touching the skin, the expected spike time difference would be −17 ms.
- **Expected spike count** was defined as the sum of spikes in response to tactile and electrical *reference* stimulation, detected within 50 ms after the stimulus onset.
- **Membrane potential shift** (mV) was defined as the difference between the median membrane potential measured during a 1,000 ms period before hyperpolarization and the 200 ms period during hyperpolarization directly before the tactile stimulation was applied (Figure 1G).
- **Q**_**10**_ was defined as the factor of the first spike latency at higher temperature divided by the first spike latency at lower temperature, given a temperature increase of 10°C (Eberhard et al., 2015). Therefore, a Q_10_ smaller than 1 indicates shorter latencies for higher temperatures.
- **Latency difference to warmest trial** (ms) was defined as the difference between the first spike latency in a given trial minus the latency in the warmest trial in response to identical stimulation in the same experiment.
- **Temperature difference to warmest trial** (°C) was defined as the temperature difference of a given trial and the warmest trial in that experiment.

We tested the differences between the medians of two distributions with the non-parametric Wilcoxon signed-rank test to account for repeated – and therefore dependent – measures (applied in sections 3.1 and 3.4, and in Supplementary Figure S2; α = 0.05; Wilcoxon, 1945). Bonferroni correction was applied to adjust the significance level to α = 0.025 when comparing Q_10_ values of responses to electrical and tactile stimulation to 1 and to each other (multiple testing, section 3.4). We calculated Spearman’s rank correlation (*r_s_*) to characterize the relationship between two measures and tested the slope of the fitted line against zero to test for statistical significance (sections 3.2 and 3.4; α = 0.05). All *p*-values and test statistics are rounded to the second decimal place. *p*-values below 0.01 are deemed significant and indicated as *p* < 0.01.

**Figure 3:**
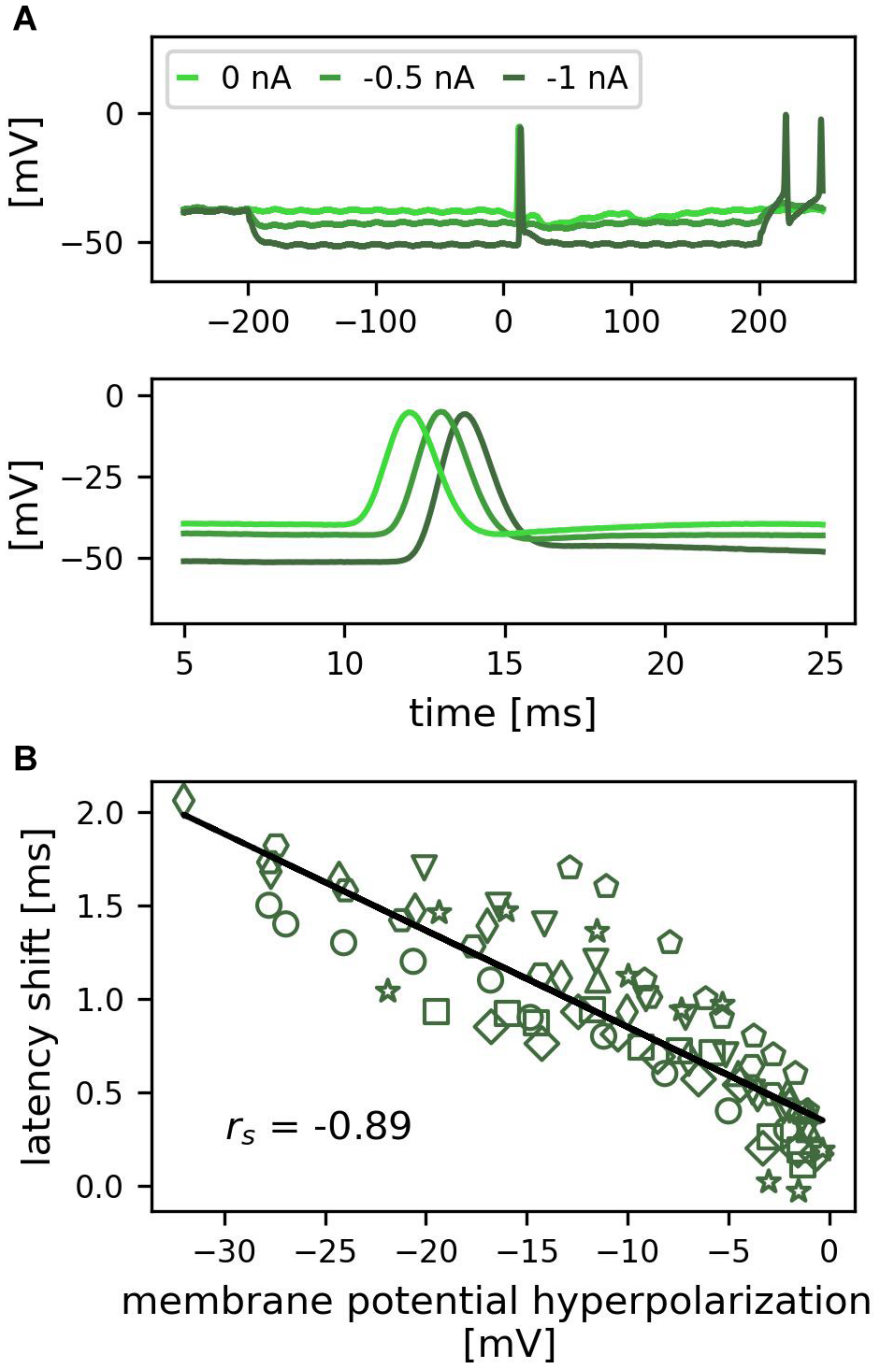
Hyperpolarization of the soma delays first spike latencies of tactilely induced spikes. **(A)** Example recording of one trial for three different amplitudes of negative current injection (0, −0.5, −1 nA). The top panel shows a 500 ms period including the 400 ms of somatic hyperpolarization, the bottom panel shows an enlarged detail of the 20 ms period, during which the spikes from the skin reached the soma. **(B)** Correlation between the hyperpolarization of the membrane potential by negative current injection and the first spike latency shift across cells (*n* = 10 cells, individual cells indicated by different symbols, 1 trial; *r_s_* = −0.89, *p* < 0.01).

## 3. Results

This study investigates the interaction of action potentials which are generated by the same neuron at two different locations that are (depending on the size of the animal) between 5 and 10 millimeters apart (Figure 1A). All experiments were carried out in leech body-wall preparations (Figure 1B), allowing us to mechanically stimulate the skin while electrically stimulating and recording from the mechanosensory T cell soma at the same time. Tactile stimulation elicited spikes in the skin that were propagated over a distance of up to 10 mm until they reached the soma. Electrical stimulation triggered spikes somewhere close to the soma, but the exact location of this central spike initiation zone is unknown (Figure 1A).

### 3.1. First spike latencies of spikes elicited in the skin are more precise than of spikes elicited near the soma

We performed intracellular recordings from T cells while stimulating the skin. Spikes were triggered either via touching the skin with a *standard tactile pulse* (5 ms duration, 8 mN amplitude), via depolarization of the soma with a *standard electrical pulse* (5 ms duration, +1.5 nA amplitude), or via releasing the soma from hyperpolarization (400 ms duration, −1 nA amplitude) (Figure 1). Figure 2A presents an example recording of one T cell with one trial highlighted and nine additional trials superimposed, which shows that the three types of stimulation triggered spikes with different precision and reliability. The positive current injection of the *standard electrical pulse* into the soma reliably elicited one spike in every trial precisely after a short latency of in this example between 5.4 ms and 6.1 ms (top panel; red). Across T cells, each *standard tactile pulse* triggered one to four spikes that reached the recording electrode in the soma, while each preparation yielded a reliable spike count to the repeated *reference* tactile stimulation. The example cell in Figure 2A reliably elicited two spikes in all trials (middle panel; green). They reached the soma precisely timed with first spike latencies ranging from 13.5 ms to 13.9 ms. In contrast, the release of negative current injection elicited in this example recording rebound spikes only in two of the ten trials and with first spike latencies of 27.4 ms and 29.6 ms, respectively (bottom panel; black). Figure 2B shows the phase plane trajectories of spikes elicited with the three different stimulation types for the highlighted trial in Figure 2A. While the trajectories of tactilely induced spikes (green) and rebound spikes (black) resemble each other, the passive response to positive current injection (red) shifts the trajectory of the spike to a more depolarized starting point. In contrast to the spike depolarization phase, the spike afterhyperpolarization trajectories look virtually identical for all three types of responses, because the passive response to the brief *electrical pulse* of 5 ms already ceased before the spike afterhyperpolarization started.

Across cells, the reliability was 98.85% for spikes elicited by positive current injection into the soma (*n* = 960 data points), and 99.9% for spikes elicited in the skin (*n* = 960 data points). The release of a hyperpolarization of −1 nA elicited rebound spikes with a probability of only 51.25% (*n* = 240 data points). We therefore focused all further analyses on first spike latencies elicited via *the standard tactile and electrical pulses*. Figure 2C shows the median latencies of *n* = 24 cells for spikes elicited by tactile versus electrical stimulation of the fourth *reference*. At room temperature, the median latency of the first spikes in response to the *standard electrical pulse* ranged from 5–9 ms between cells, with a median of 5.7 ms. First spike latencies of spikes induced by the *standard tactile pulse* were significantly longer (*z* = 4.29, *p* < 0.01), because spikes traveled several millimeters from the skin to the cell body in the ganglion (Figure 1A). In tactile responses, latencies depended on the touch location relative to the cells’ receptive field and therefore varied considerably across cells, with a range of 11–28 ms and a median of 17.2 ms. Figure 2D shows the latency ranges of electrically and tactilely induced spikes within cells for the fourth *reference* (*n* = 24 cells, 10 trials). For spikes induced by the *standard electrical pulse*, repeated stimulation of the same cell yielded a median latency range of 1.1 ms between the longest and the shortest latency. However, with a median latency range of 0.4 ms, the temporal precision of spikes elicited in the skin by the *standard tactile pulse* was significantly higher (*z* = 2.8, *p* < 0.01). In conclusion, spikes induced by touch to the skin, as well as by depolarization of the soma were both elicited reliably by all stimulus presentations, in contrast to rebound spikes. Moreover, spikes elicited in the skin reached the soma temporally more precise than spikes triggered by somatic current injection.

### 3.2. Somatic hyperpolarization increases the first spike latency of tactilely induced spikes

Since T cells receive inhibitory synaptic inputs from unknown cells in the isolated ganglion (Scherer et al., 2022), we mimicked the inhibitory synaptic currents by hyperpolarizing the soma with negative current steps of 400 ms duration and amplitudes ranging from −0.1 to −1 nA in steps of 0.1 nA, in combination with applying a *standard tactile pulse* to the skin (Figure 1G). Figure 3A shows the responses to three negative current steps in an example recording. In each of the conditions, one spike was elicited by the tactile stimulation at time 0. At resting membrane potential (light green), the latency of this spike was 12.6 ms (see lower panel in A for the exact timing). When the soma was hyperpolarized, the propagation time from the skin to the soma increased, leading to a latency of 13.7 ms for −0.5 nA (green) and 14.9 ms for −1 nA (dark green). Therefore, the stronger the cell was hyperpolarized during tactile stimulation, the longer was its spike propagation time and the latency of spikes recorded in the soma. This systematic change also holds true across cells (Figure 3B, different markers indicate individual cells). The correlation between the change in membrane potential and the first spike latency shift was statistically significant (*n* = 10 cells, *n* = 95 datapoints; *r_s_* = −0.89, *p* < 0.01). A hyperpolarization of −20 mV shifted the first spike latency by approximately 1 ms. Hence, even though the spikes were generated in the skin at a distance of approximately 5–10 mm, their propagation was strongly influenced by the membrane potential in the central compartments of the neuron, leading to a correlation between the membrane potential hyperpolarization and the arrival time at the soma.

### 3.3. Spikes elicited by tactile and electrical stimulation interact

**Figure 4:**
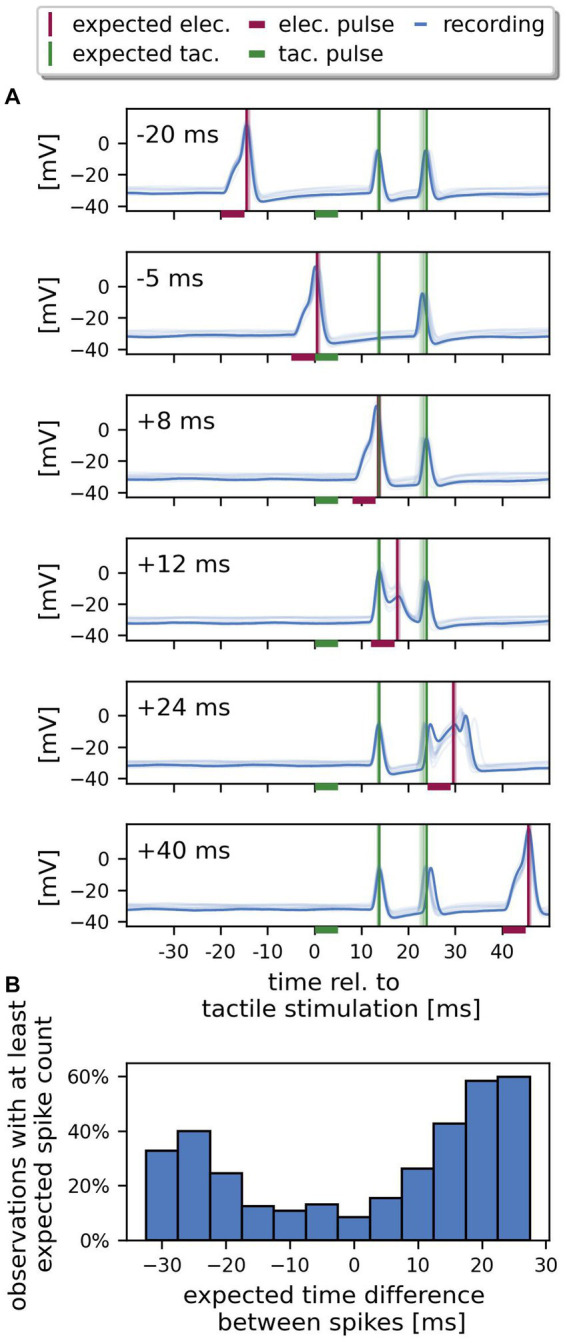
Spikes from both SIZ can interact. **(A)** Example recording of one cell. The recorded responses (blue) to ten repetitions of identical stimulation are superimposed (one selected trial in dark colors, the other 9 trials in lighter colors). Each panel shows the responses to stimulation with a different time delay between *electrical* and *tactile pulse*, ranging from −20 ms to +40 ms. The horizontal bars indicate the stimulus period, the vertical lines in each panel mark the expected timing of spikes elicited by *electrical pulses* (red) and by *tactile pulses* (green), if their response latencies would be identical to the responses to the *reference* stimuli. At −20 ms (electrical before tactile stimulation) and + 40 ms (electrical after tactile) delay, this cell responded with one spike to the somatic current injection and with two spikes to the *tactile pulse*. For a delay of −5 and + 8 ms, only one spike from the skin was recorded consistently over trials. For a delay of +12 ms, the *electrical pulse* failed to elicit a spike. At +24 ms delay, the spike elicited by the *electrical pulse* is detected after the second spike triggered by the *tactile puls*e and is delayed compared to the expectation based on response to the *reference* stimulation. **(B)** The percentage of observations with at least the expected spike count depends on the expected time difference between the response to the tactile and the *electrical pulse* (*n* = 24 cells, 10 trials, 4,727 data points). Only bins with at least 150 observations are shown. Negative expected spike time differences indicate that the spike elicited via current injection to the soma was recorded before the spike elicited by touching the skin, and vice versa.

It is known that T cells receive both inhibitory and excitatory synaptic inputs from other unknown cells in the ganglion, which can cause spikes at the SIZ near the soma in T cells (Baylor and Nicholls, 1969; Burgin and Szczupak, 2003). Additionally, tactile stimulation triggers spikes at the SIZ near the skin. We therefore investigated potential interactions of the two SIZ by eliciting single spikes via current injection of the *standard electrical pulse* (5 ms duration, +1.5 nA amplitude) to the soma in combination with mechanical stimulation of the skin by the *standard tactile pulse* (5 ms duration, 8 mN amplitude). In the example presented in Figure 4A, the cell reliably elicited two spikes in response to the tactile stimulation. We systematically varied the stimulus timing of the *electrical pulse* relative to the *tactile pulse* from −20 ms to +40 ms (Figure 1F).

Figure 4A shows an example recording of one cell with ten trials superimposed (one trial highlighted by darker color) for six different relative timings between *tactile* and *electrical pulse*. We compared the timing of the recorded spikes to the spike times that were expected based on the corresponding *references* protocol (vertical lines in Figure 4A, green: tactile, red: electrical stimulation). In the *references*, the stimulation pulses were separated by 1 s and therefore could not interfere with each other. When both stimulation pulses were separated by a long delay (−20 ms or + 40 ms), the *electrical pulse* (red horizontal bar) reliably elicited one spike with a latency of 5.8 ms in the highlighted trial, while the *tactile pulse* (green horizontal bar) reliably caused two spikes with a first spike latency of 13.5 ms, matching the expected spike times perfectly. The timing of the second spike triggered by the *tactile pulse* was more variable over trials (broader range of light green vertical lines) than the first spike. For smaller delays between both stimulations (−5, +8, and + 12 ms) the total number of detected spikes was reduced. For a delay of −5 ms, the spike elicited by the *electrical pulse* shortly before the application of the *tactile pulse* blocked the expected first tactilely induced spike from reaching the soma, while the second spike was recorded approximately at the expected time. When both the response to the *electrical pulse* and the first spike elicited by the *tactile pulse* were expected to arrive in the soma at the same time (+8 ms stimulus delay, red and green vertical line match in time), again only two spikes were recorded consistently over trials. The single spike at the time expected for both types of stimulation could indicate that one spike was generated at each of both SIZ, but both spikes merged before reaching the soma, or one spike was slightly earlier and blocked the other. Again, the second spike reached the soma approximately 24 ms after the tactile stimulation as expected from the *references*. For a delay of +12 ms, both tactilely induced spikes reached the soma as expected, but the *electrical pulse* failed to elicit a spike (the small peak at the expected spike time corresponds to the end of the passive response to the *electrical pulse*). At a delay of +24 ms between both pulses, a current-induced spike is detected in the soma (confirmed by spike amplitude, spike shape and after-hyperpolarization) but with an increased first spike latency compared to the response to the corresponding *reference* (red horizontal line). Figure 4B shows that the percentage of observations, in which the sum of detected spikes was at least equal to the expected spike count, depended on the expected spike time difference. Given the null hypothesis that spikes from both SIZ do not interact, the percentage of observations with the expected number of spikes should be independent of the spike time difference. However, as Figure 4B shows, for large positive or negative spike time differences, the number of observations with at least the expected spike count was higher than for smaller differences, when the spikes were expected to arrive at the same time or shortly after each other. The resulting dip in the histogram near 0 ms indicates that fewer spikes were detected in the soma when the spikes were expected to arrive in temporal proximity. This finding confirms the observation in Figure 4A, where the sum of spikes is three for long delays (−20, +24, and + 40 ms) but decreases to two for smaller expected spike time differences (−5, +8, and + 12 ms). It should be noted that for many cells in our sample even expected time differences as long as 30 ms decreased the spike count compared to the responses to the independently delivered pulses in the *references.* In conclusion, spikes from both SIZ interacted – spikes from both origins can suppress each other, or the spike arrival time in the soma can be shifted by preceding spikes.

### 3.4. Temperature influences first spike latencies from both SIZ

**Figure 5:**
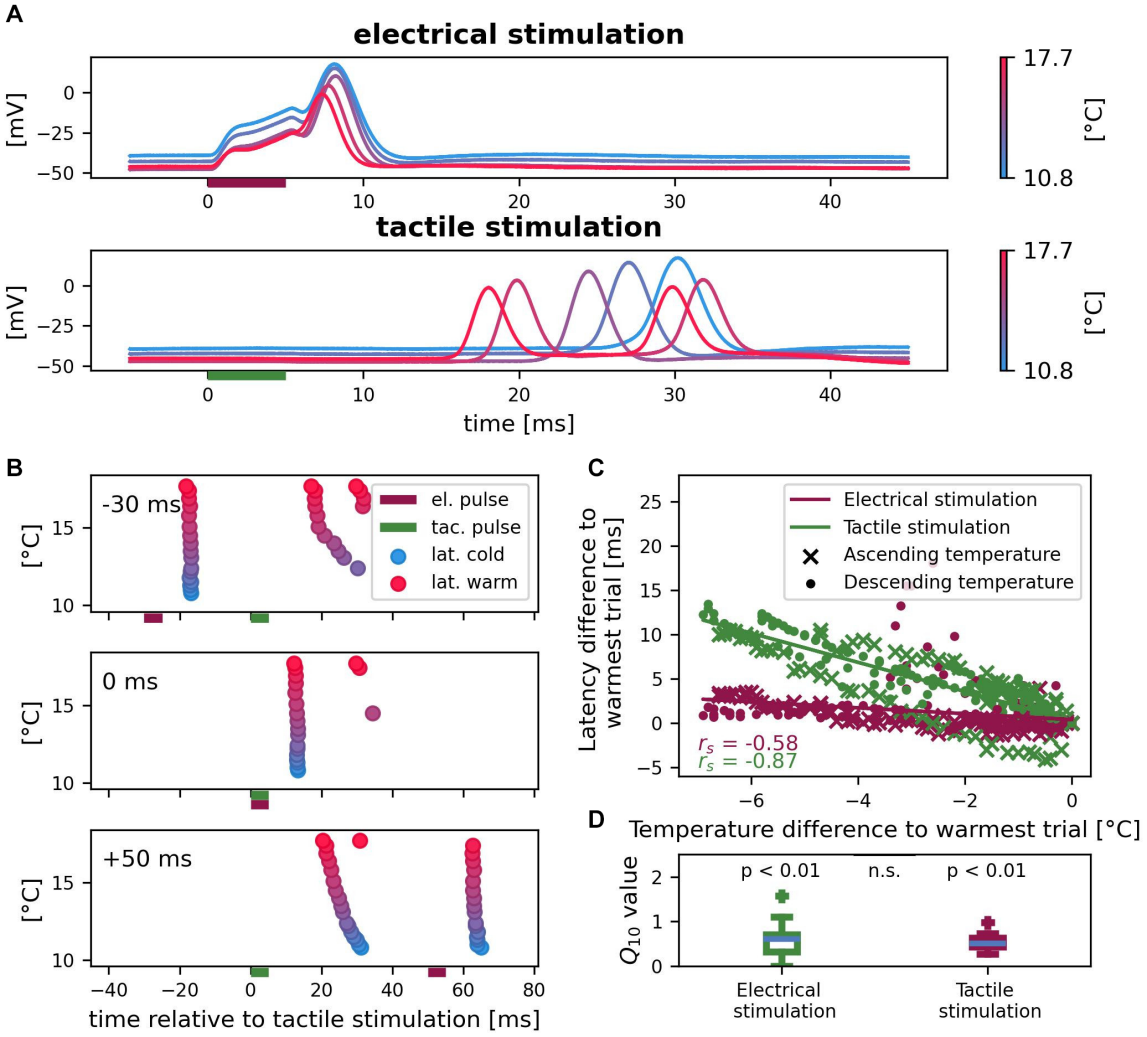
Effects of temperature on spike times of electrically and tactilely induced spikes. **(A)** Example recording of one cell with five selected trials in different temperature conditions. Vertical bars indicate the timing of the *electrical* (red) and *tactile* (green) *stimulation pulse* in a *reference*. **(B)** Spike times for all 17 trials of the same cell as in **(A)** with different temperatures and three different sequences of electrical (red horizontal bar) and tactile (green horizontal bar) stimulation. Color scale of temperature as in **(A)**. **(C)** Correlation between temperature and latency differences to the warmest trial for spikes elicited by *tactile pulses* in the skin (green) and by *electrical pulses* near the soma (red) in the *references*. Since absolute latencies and temperature conditions differed across cells, both are normalized to the warmest trial for each cell (*n* = 14 cells, 10 with ascending and 4 with descending temperature, with *reference* stimulus #4 in different numbers of trials; 144 data points). **(D)** Q_10_ values of first spike latency changes differ from 1 (null hypothesis no change with temperature) both for electrical and for tactile stimulation (both *p* < 0.01), but do not differ significantly between the responses to both types of stimulation (*p* = 0.68; same data as in **C**).

We investigated the effects of temperature on first spike latencies of tactilely and electrically induced spikes applying the *temperature* protocol. We continuously recorded intracellularly while changing the temperature of the preparation. All *n* = 14 experiments together covered a temperature range of 7.9 – 23.9°C, but individual temperature ranges varied. *n* = 10 experiments were performed under ascending and n = 4 experiments under descending temperature conditions. Figure 5A shows an example recording of one cell in varied temperature conditions. The lower the temperature was, the higher and broader were spikes from both origins. First spike latencies measured in the soma consistently increased for lower temperatures for both types of stimulation. While the latency difference between the warmest and the coldest condition was 0.8 ms for responses to somatic current stimulation, it differed by as much as 12.2 ms for the spikes that propagated from the skin to the soma. Additionally, tactile stimulation triggered two spikes in higher temperatures (red), but only one spike in cold conditions (blue) in this example cell. Figure 5B shows in a raster plot the development of spike times of the same cell for different temperatures, when *tactile* and *electrical pulses* were applied in combination. Again, a higher temperature consistently caused shorter latencies of spikes reaching the soma. This experiment demonstrates that depending on the order of stimulation, higher temperatures can decrease (−30 ms delay) or increase (+50 ms delay) the relative timing between spikes from both origins, because of the greater effect of temperature on the propagation of spikes from the skin. Additionally, temperature can influence the spike count, with second spikes arriving from the skin only at higher temperatures for this example cell. When spikes were elicited by both types of stimulation in temporal proximity (0 ms delay), fewer spikes were detected in the soma. This spike interaction effect, which we already described in section 3.3 for room temperature, was found consistently for the entire temperature range. Figure 5C shows that this relationship between temperature and first spike latency shift holds true across cells and for ascending as well as descending temperature conditions. Latencies of both electrically and tactilely induced spikes were influenced by temperature changes, but the total decrease in latency with increasing temperature was stronger for spikes propagating from the skin to the soma (electrical: *r_s_* = −0.58, *p* < 0.01; tactile: *r_s_* = −0.87, *p* < 0.01). A temperature change of 1°C shifted the first spike latency of spikes from the central SIZ by approximately 0.3 ms, for spikes from the SIZ in the skin by approximately 2 ms. The negative correlation coefficient as well as Q_10_ values smaller than 1 shown in Figure 5D indicate shorter latencies for higher temperatures. Comparing the distributions of Q_10_ values of first spike latency changes reveals that the stronger temperature effect on the latencies of tactilely induced spikes was only due to the larger absolute propagation times of these spikes compared to the local responses to somatic current injection. In contrast, the relative differences in latency measured as Q_10_ did not differ significantly between both origins of spikes. The median Q_10_ values were 0.60 for electrical stimulation and 0.51 for tactile stimulation and both differed significantly from 1 (electrical: *z* = 2.61, *p* < 0.01; tactile: *z* = 3.30, *p* < 0.01), but not from each other (*z* = 0.41, *p* = 0.68). In conclusion, temperature influences the latency of spikes elicited by both types of stimulation with the same factor. However, since spikes from the skin travel longer distances to reach the soma (Figure 1A), their higher absolute propagation times also lead to longer delays in spike times for colder temperatures than observed for spikes that were elicited close to the soma.

## 4. Discussion

In this study, we showed that at least three different types of stimulation can trigger spikes in the mechanosensory T cell of the leech: somatic depolarization, the release of somatic hyperpolarization and touch applied to the skin (Figures 2A,B). This is consistent with the conclusion from prior experimental and modeling studies that T cells have a spike initiation zone close to the soma that responds to synaptic inputs, in addition to the spike initiation close to the skin in response to tactile stimulation (Burgin and Szczupak, 2003; Kretzberg et al., 2007). While it is evident that the two SIZ are separated by 5–10 mm in adult leeches (Figure 1A), their exact locations remain to be determined. We found that latencies of spikes elicited in the skin vary considerably between cells. This is because latencies depend on the exact touch location relative to the cell’s receptive field (Pirschel and Kretzberg, 2016). We did not restrict our analysis to a specific T cell, because we found that not only the T cell with the lateral receptive field, but all three ipsilateral T cells responded to touch at 90° with different latencies (Supplementary Figure S1). This finding indicates that their receptive fields might be more overlapping than previously reported (Nicholls and Baylor, 1968; Yau, 1976; Blackshaw, 1981). Despite the wide range of latencies across preparations, repetitive touch at the same location of the same preparation revealed that the timing of spikes elicited in the skin is even more precise than of spikes induced by somatic stimulation (Figure 2D). This is consistent with previous findings on temporal encoding of stimulus location in T cells (Pirschel and Kretzberg, 2016), which suggested that T cells play a role in the local bend reflex, with which leeches bend away locally when being touched (Kristan, 1982). Despite the relevance of spike counts and timing for sensory coding, we found in this study that both response features, spike count and first spike latency, are influenced by internal and external factors.

### 4.1. Internal factors of spatial–temporal integration of inputs influence sensory coding

Our results indicate that the timing and the number of tactilely induced spikes are massively influenced by spatial–temporal integration of sensory and synaptic inputs to the T cell. When we mimicked inhibitory synaptic input by hyperpolarizing the soma during tactile stimulation, first spike latencies of spikes elicited in the skin increased linearly with the degree of hyperpolarization (Figure 3B). This phenomenon can be explained by a moderate hyperpolarization shifting the resting membrane potential away from the spike threshold. Hence, even without activating hyperpolarization-activated currents, more sodium channels need to open, slowing down the spike propagation and resulting in a later arrival time at the soma. However, the effect of inhibitory synaptic inputs could be even stronger than estimated by our hyperpolarization experiments. Synaptic inhibition would not only hyperpolarize the resting membrane potential, but also decrease the cell’s input resistance, which could lead to spike propagation failures rather than just time delays. It is therefore plausible that our approach to mimic inhibitory synaptic potentials by somatic hyperpolarization underestimates the interaction effect of the two SIZ.

Injection of short positive current pulses triggered spikes at an unknown location close to the soma and thereby mimicked the effect of suprathreshold synaptic inputs onto the central region of the T cell. When such a spike was evoked at the central SIZ shortly before a spike arrived from the skin, the arrival time of the tactilely induced action potential at the soma was delayed, presumably because of the cell’s relative refractory period. Moreover, when we elicited spikes in both SIZ in temporal proximity, fewer spikes were detected in the soma (Figure 4B). Since we recorded intracellularly from the soma, it is impossible to tell if spikes from both origins merged when traveling along the cell membrane, or if the first spike blocked the slightly later second spike due to the absolute refractory period.

Since relative spike timings best encode the stimulus location in T cells, even small latency changes might have relevant consequences for a behaving leech (Pirschel and Kretzberg, 2016). What could be the functional benefit of these spike timing changes observed in this study? Firstly, for a wide range of stimulation intensities, all mechanosensory cell types elicit spikes (Kretzberg et al., 2016). Since T cells mainly receive inhibitory synaptic input from the other mechanosensory Pressure (P) and Nociceptive (N) cells (Burgin and Szczupak, 2003), strong tactile stimulation that activates all mechanoreceptors might delay the arrival times of spikes traveling from the skin to the T cell soma. Although the hyperpolarizing step currents of 400 ms duration used in this study are not directly comparable to the inhibitory synaptic input T cells receive from the network, they provide evidence for an influence of inhibition on spike times. We speculate that spike time delays caused by inhibition from P and N cells might help the leech T cells to modulate its mechanosensory response in a stimulus-dependent manner. In this study, however, we did not aim to analyze the interaction of different mechanoreceptor types and therefore chose a light and short touch of 8 mN to only trigger T cell spikes. Even though it was shown previously that 10 mN can be enough to elicit single P cell spikes, their response latencies would be too long to influence T cell activity during the response period we considered in this study (Pirschel and Kretzberg, 2016). Simultaneous recording from T and P or N cells combined with tactile stimulations of varied intensities could help to investigate this putative interaction between the mechanosensory cell types.

Secondly, spike timing can also be changed with a spatial shift of SIZ, which was shown to serve as homeostatic plasticity mechanism in several systems (Kuba et al., 2010; Grubb et al., 2011; Städele and Stein, 2016; Yamada and Kuba, 2016; Goldstein et al., 2019; Ha and Rasband, 2019). For example, in anterior gastric receptor neurons of the stomatogastric nervous system of *Cancer borealis*, the dislocation of the ectopic SIZ via neuromodulation abolishes the cells’ signal integration (Städele and Stein, 2016; Städele et al., 2018). In these studies, the authors also emphasize the role of backpropagating spikes for the signal integration of two spike initiation zones. Since T cells both are susceptible to neuromodulation (Gascoigne and McVean, 1991), and have backpropagating spikes (Van Essen, 1973), their influence on the interaction of spike initiation zones could be a future topic to investigate. To reach this goal, antibody staining and multi-compartment modeling will be needed to identify the SIZ locations. Once the interaction of the two SIZ are described within the T cell in more detail, voltage-sensitive dye recordings can help us to understand how the T cell spike timing affects the postsynaptic partners, such as the S cell or the local bend interneurons, e.g., 157 and 159 (Muller and Scott, 1981; Wagenaar, 2015; Pirschel et al., 2018).

### 4.2. The external factor temperature influences sensory coding

As a global factor, temperature influences nervous systems in manifold ways (Robertson and Money, 2012; Marder and Rue, 2021). Consistent with previous findings that an increased temperature decreases spike latencies (Eberhard et al., 2015), we showed that temperature influences the propagation time of tactilely and electrically triggered spikes by the same factor (Figure 5D). Hence, when the temperature decreased by, e.g., 5°C, the longer propagation time of spikes coming from the skin were delayed by several milliseconds, while the shorter latencies of spikes originating from the central part of the neuron only changed on the order of one millisecond (Figures 5A–C). Consequently, the relative timing between spikes from both origins depends critically on temperature. While the seasonal variations in room temperature ranged 17–25°C between the experiments in this study, a temperature change of about 0.5° C was sufficient to increase the propagation time of spikes elicited from the skin to the soma by about 1 ms (Figure 5C). In comparison, a − 20 mV hyperpolarization of the soma was needed to reach the same effect (Figure 3B), which is approximately four times higher in amplitude than the inhibitory synaptic input that an unstimulated T cell receives from the network (Alonso et al., 2020; Scherer et al., 2022).

During their lifetime, leeches encounter in their natural habitat temperatures ranging from a frozen pond to a shallow puddle heated up by the bright summer sun. Moreover, a swimming leech can move between water regions with temperature differences of several degrees within minutes. Hence, the survival of this poikilothermic animal depends on the ability to robustly perceive and react to tactile stimuli under dynamic temperature conditions. As a global factor, temperature influences many biophysical processes in the intact organism (Robertson and Money, 2012; Marder and Rue, 2021). Most probably, also the spike times of the other mechanoreceptor types and their synaptic input onto the T cell depend on temperature. Hence, it remains to be investigated how sensory coding based on spike times that results from the spatial–temporal interaction of sensory and synaptic inputs can lead to robust behavior. The described effect of temperature on spike times might be counterbalanced or enhanced by other temperature-dependent processes in a behaving leech, which require more holistic approaches to be investigated.

### 4.3. Outlook

The mechanosensory T cell in the leech is a well-suited model system to study the integration of diverse inputs from multiple SIZ in sensory neurons. Spikes can be triggered experimentally both in the skin and near the soma reliably with a high temporal precision. We used this opportunity to show that the propagation time of spikes from the skin is modulated by inhibitory synaptic inputs, by spikes elicited near the soma and by temperature. Hence, concerning the spike responses measured in the soma, the two SIZ in T cells do not act as independent compartments.

Moreover, the potential of the leech T cells as a model system goes beyond this biophysical perspective. Despite the small number of cells in their nervous system, leeches can discriminate between touch locations as well as the human fingertip (Baca et al., 2005) and more precisely than what can be explained solely by the activity of the sustained responses of P cells (Thomson and Kristan, 2006). Therefore, it was suggested that the local bend network relies also on the relative spike timing of T cells with overlapping receptive fields to localize tactile stimulation (Pirschel and Kretzberg, 2016) by giving input to local bend interneurons (Kretzberg et al., 2016; Pirschel et al., 2018). Voltage-sensitive dye recordings show the impact of T cell activity on the network (Fathiazar et al., 2018), and can reveal how the interaction of tactilely induced spikes and synaptic inputs to the T cell influences the reaction of interneurons. Hence, the combination of experimental and modeling techniques can elucidate the role of the mechanosensory T cell responses that are modulated by synaptic inputs from the network, as well as by external factors like temperature. Therefore, the leech might serve as a key to the fundamental question how poikilothermic organisms can use temporal coding to generate adequate behavioral reactions to sensory stimulation in changing environmental conditions.

## Data availability statement

The datasets generated for this study are publicly available at: https://gin.g-node.org/Computational_Neuroscience_UOL/Scherer_et_al_2023.

## Ethics statement

Ethical approval was not required for the study involving animals in accordance with the local legislation and institutional requirements because German law only requires ethical approval for experiments with tissue of vertebrates and higher invertebrates.

## Author contributions

JSS and JK: conceptualization and drafting manuscript. JSS and BS: data collection. KS: data analysis. JSS, KS, BS, and JK: interpretation and reviewing and editing manuscript. JK: project administration and supervision. All authors contributed to the article and approved the submitted version.

## Funding

We thank the DFG for financial support by Germany’s Excellence Strategy Cluster EXC 2177/1 “Hearing4all”, as well as project KR 2925/4-1. KS was supported by Studienstiftung des Deutschen Volkes.

## Conflict of interest

The authors declare that the research was conducted in the absence of any commercial or financial relationships that could be construed as a potential conflict of interest.

## Publisher’s note

All claims expressed in this article are solely those of the authors and do not necessarily represent those of their affiliated organizations, or those of the publisher, the editors and the reviewers. Any product that may be evaluated in this article, or claim that may be made by its manufacturer, is not guaranteed or endorsed by the publisher.
