# Supplementary material for: Synaptic input and temperature influence sensory coding in a mechanoreceptor
Source: Front Cell Neurosci. 2023 Sep 12;17:1233730. doi: 10.3389/fncel.2023.1233730 (PMC10522859; doi:10.3389/fncel.2023.1233730)
Supplement: Supplementary file 1 [file Image_1.pdf]

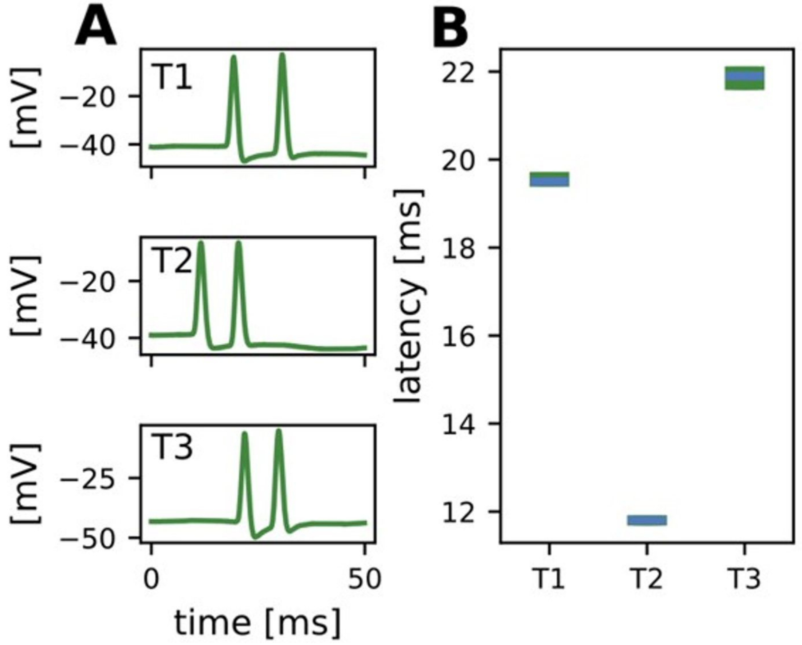

Supplementary Figure 1: Tactile stimulation at the same location (90°) elicited spikes in all three ipsilateral T cells. **(A)** Recordings of the three T cells were performed in the same ganglion in succession (single cell intracellular recordings). **(B)** Boxplots of first spike latencies of the same cell for ten trials.
