# Supplementary material for: Synaptic input and temperature influence sensory coding in a mechanoreceptor
Source: Front Cell Neurosci. 2023 Sep 12;17:1233730. doi: 10.3389/fncel.2023.1233730 (PMC10522859; doi:10.3389/fncel.2023.1233730)
Supplement: Supplementary file 2 [file Image_2.pdf]

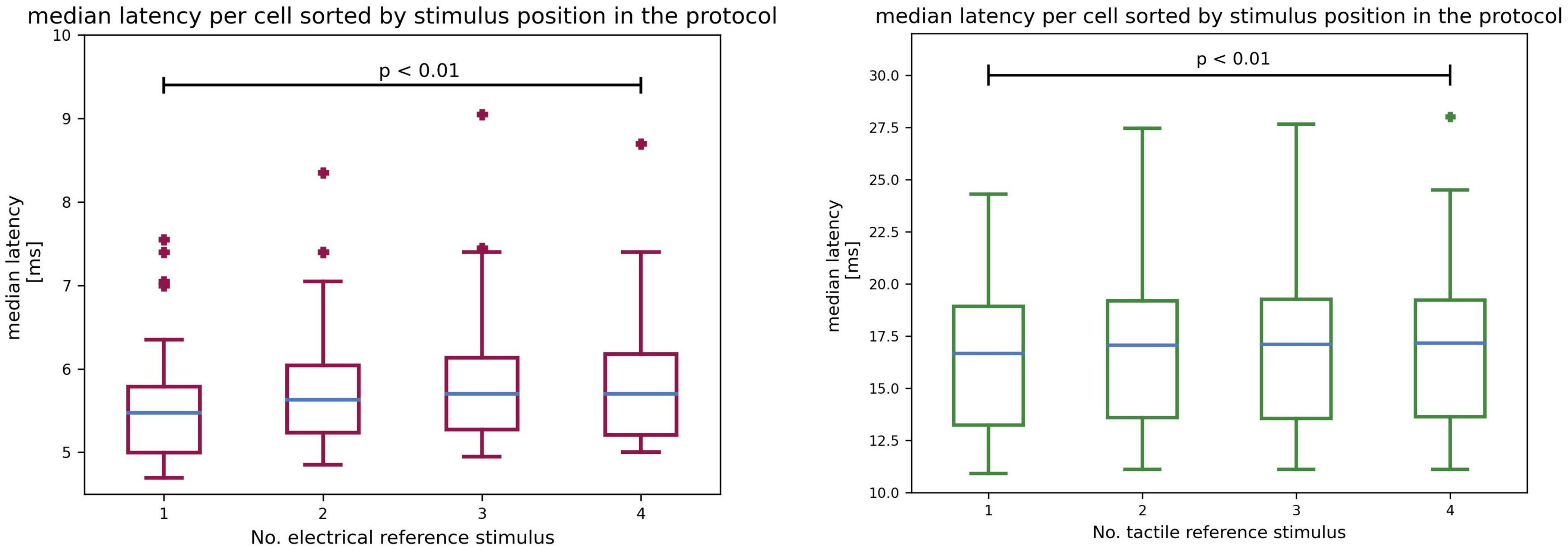

Supplementary Figure 2: first spike latencies of both spikes elicited in the skin and in the soma increased over the course of the four *reference* stimuli (electrical: $z = 4.88$, $p < 0.01$, tactile: $z = 3.94$, $p < 0.01$).
